# Mild One-Step Protein Recovery from Microalgae Cultivated in Swine Wastewater Using Natural Deep Eutectic Solvent-Based Aqueous Biphasic Systems

**DOI:** 10.3390/molecules31030483

**Published:** 2026-01-30

**Authors:** David Moldes, Marisol Vega, Silvia Bolado, Patricia F. Requejo

**Affiliations:** 1 Institute of Sustainable Processes, University of Valladolid, 47011 Valladolid, Spain; david.moldes@uva.es (D.M.); mariasol.vega@uva.es (M.V.); silvia.bolado@uva.es (S.B.); 2 Department of Analytical Chemistry, Faculty of Sciences, University of Valladolid, 47011 Valladolid, Spain; 3 Department of Chemical Engineering and Environmental Technology, School of Industrial Engineering, University of Valladolid, 47011 Valladolid, Spain

**Keywords:** aqueous two-phase system (ATPS), protein recovery, green extraction

## Abstract

Photobioreactor-based microalgae cultivation offers an integrated approach for nutrient-rich wastewater treatment while producing valuable biomass. One of the main microalgae components is proteins, making them a biotechnological target. In this work, to develop efficient and greener extraction methodologies, aqueous two-phase systems (ATPSs) based on natural deep eutectic solvents (NADESs) were evaluated for one-step protein extraction from microalgae cultivated in swine wastewater. Six ATPSs combining two NADES—betaine:levulinic acid (Bet:2LA) and choline chloride:urea (ChCl:2Urea)—and their individual components (Bet or ChCl) with phosphate salts were compared. Systems {NADES + K_3_PO_4_ + water} were characterized and reported for the first time. Protein recovery yield (PRY) and selectivity (protein-to-carbohydrate mass ratio, R) were assessed for three extraction times and at room temperature. The ATPS {Bet:2LA + K_3_PO_4_ + H_2_O} achieved a PRY of 16.4% and remarkable selectivity after 30 min (R = 2.17 g·g^−1^), with proteins concentrated in the NADES-rich phase, and negligible recovery in the salt-rich phase. Although the maximum PRY (18.2% at 120 min) was achieved with the precursor betaine, the ATPS with Bet:2LA at 30 min offered an optimal balance between efficiency and process time. With a water content of up to 50%, these systems underscore the potential of NADES-based ATPSs as sustainable platforms for protein recovery.

## 1. Introduction

Water pollution remains one of the most pressing global concerns, and one which is intensified by rapid population growth, intensive livestock farming, and aquaculture [1,2]. Microalgae-based photobioreactors have emerged as a sustainable and efficient alternative for conventional wastewater treatment, especially on farms [3], as they effectively mitigate pollutant loads while generating biomass rich in valuable compounds by cultivating microalgae in symbiosis with heterotrophic and nitrifying bacteria [4,5]. Proteins comprise 30–60% of this dry biomass [6], establishing the economic feasibility of their recovery and use in diverse applications, from functional polypeptides to agricultural biostimulants, or for animal feed [7]. Despite this potential, the main challenges hindering industrial-scale implementation lie in the efficient extraction and purification of these compounds from the complex microalgal matrix [8].

The conventional processes used for protein recovery often involve multiple steps, including physical, chemical, or enzymatic pretreatments (e.g., high-pressure homogenization, bead milling, alkaline or enzymatic hydrolysis, or thermal processing) followed by extraction using toxic and non-renewable solvents (e.g., hydrocarbons or alcohols) [9]. These methods are typically energy-intensive, produce hazardous waste requiring costly waste management, or employ harsh conditions that can degrade the proteins [10]. To address these concerns, aqueous two-phase systems (ATPSs) have gained increasing attention as a promising alternative to conventional separation and purification methods as, in addition to their simplicity and eco-friendly characteristics, they provide mild conditions that do not harm or denature the extracted biomolecules [11]. These systems are composed of water, a salting-out agent, and an additional component that, when mixed in appropriate proportions, form two immiscible aqueous phases [12], which present distinctive properties, enabling the selective partitioning and recovery of different biomolecules, like proteins, antibodies, or hormones, based on their relative affinities [13,14]. The use of deep eutectic solvents (DESs) in ATPSs has emerged as a promising strategy to further enhance sustainability and tunability [13]. DESs are composed of one or more hydrogen bond acceptors (HBAs) and one or more hydrogen bond donors (HBDs) that, when mixed in the proper molar ratio, cause the eutectic point to drastically decrease [15]. DESs are often referred to as “designer solvents” because they can be formulated to achieve specific physicochemical properties, depending on their starting components or on their molar ratios. Moreover, when DESs are formulated from natural metabolites like choline chloride, urea, betaine, or sugars, they are commonly referred to as natural deep eutectic solvents (NADESs). These metabolites are naturally present in all cell types and organisms, thereby avoiding drawbacks such as toxicity and environmental risks, while combining the attractive properties of DESs [16]. Moreover, NADESs have demonstrated excellent performance as extraction agents due to their high solubilizing power and biomolecule compatibility [17]. It is worth noting that these ATPSs are pseudoternary systems {NADES + salt + water}, which allows for fine-tuning of the separation process [18].

Therefore, the development of ATPS formulations based on biodegradable compounds for protein extraction is of significant interest. To date, most studies on microalgae protein recovery have relied on ionic liquids or conventional polymer–salt systems. These studies often focus on purified proteins or simplified biomass matrices and typically require energy-intensive pretreatment steps for cell disruption [19]. Although NADES-based ATPSs have emerged as greener alternatives, their application to protein extraction remains limited and has not yet been extended to microalgal biomass [20,21,22,23]. Consequently, there is a knowledge gap regarding the performance and selectivity of NADES-based ATPSs for protein recovery from complex microalgal biomass cultivated in wastewater, particularly under mild conditions and without mechanical pretreatment.

In this context, this study evaluates the potential of NADES-based aqueous two-phase systems combined with phosphate salts for the selective recovery of proteins from *Scenedesmus almeriensis* biomass cultivated in photobioreactors treating pig slurry wastewater. Specifically, the extraction performance of six ATPS—comprising two NADES and their individual hydrogen-bond acceptors—is assessed across various extraction times. The goal is to maximize protein recovery while minimizing carbohydrate co-extraction through a one-step extraction–purification strategy. By integrating environmentally friendly solvents with microalgal biomass valorization, this work advances scalable and sustainable biorefinery processes aligned with the principles of green chemistry and the circular bioeconomy.

## 2. Results and Discussion

### 2.1. ATPS Screening

As the first step of this research, an initial study was conducted to assess the feasibility of forming ATPSs, at 25 °C and atmospheric pressure, using the selected natural metabolites and their NADESs combined with different salts. These natural metabolites and NADESs were selected because ATPSs based on choline chloride (ChCl), betaine (Bet), urea, levulinic acid (LA), or on the NADES ChCl:2Urea, have previously demonstrated potential for protein extraction in other matrices [24,25,26]. The NADES Bet:2LA was chosen due to its widespread use and promising extraction performance, as reported previously [20]. Regarding the salting-out agents, both inorganic and organic salts were tested: potassium phosphate (K_3_PO_4_), potassium hydrogen phosphate (K_2_HPO_4_), trisodium citrate (Na_3_C_6_H_5_O_7_), disodium L-(+)-tartrate (Na_2_C_4_H_4_O_6_), and potassium sodium tartrate (KNaC_4_H_4_O_6_). These salts have been extensively used in similar systems according to the literature [27,28]. The results are depicted in Table 1.

As can be observed, the combination of urea and levulinic acid with the selected salts did not lead to the formation of the two distinct liquid phases necessary for the formation of an ATPS under the analyzed conditions. As for the other compounds tested, K_3_PO_4_ was the most effective salting-out agent, forming an ATPS with two natural metabolites (ChCl and Bet) and the two tested NADESs (ChCl:2Urea and Bet:2LA). The other salt forming ATPS, K_2_HPO_4_, did not lead to two immiscible liquid phases with Bet:2LA. This difference is likely due to the salting-out capacity of the salts, as the trivalent phosphate anions (PO_4_^3−^) in K_3_PO_4_ exert a stronger phase-separation effect than the divalent anions (HPO_4_^2−^) in K_2_HPO_4_. This trend aligns with the Hofmeister series, which ranks ions according to their ability to induce phase separation [32,33]. It is true that the system {Bet + K_2_HPO_4_ + water} is well described in other works [30], but it was disregarded in this work as its NADES counterpart {Bet:2LA + K_2_HPO_4_ + water} was not formed, and this work focuses on applying primarily NADES-based ATPSs. Conversely, none of the citrate and tartrate salts tested produced two immiscible liquid phases with any of the compounds used. These results are consistent with previous findings, which report that organic salts generally possess a lower phase-separation capacity than their inorganic counterparts [34].

### 2.2. ATPS Characterization

The phase diagram (binodal curve and tie-lines) of the ATPS is highly tailored to a system within specific conditions, illustrating the potential operational range of the ATPS. Examining the binodal curves and tie-lines is crucial for extracting significant details regarding component concentrations for the formation of two phases and the composition of both the top and bottom phases [35].

#### 2.2.1. Binodal Curves

**Figure 1:**
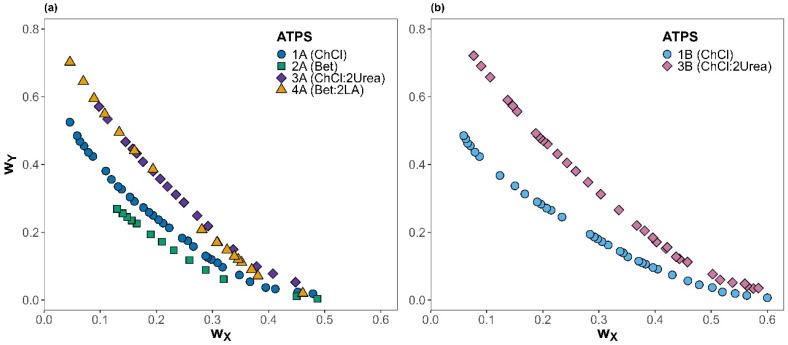
Binodal curves for ATPSs with different salts (X component) at 25 °C and atmospheric pressure: (**a**) K_3_PO_4_ and (**b**) K_2_HPO_4_. Different shapes and colors represent each system studied; the component Y is shown in brackets.

Experimental binodal curve data for the six selected ATPSs {Component Y (1) + Component X (2) + water (3)}, with natural metabolite or NADES as the selected options for Component Y, the phosphate salts as Component X, and water, determined at 25 °C and atmospheric pressure are shown in Figure 1 (data available in Supplementary Material). Although ATPSs are ternary systems, they are commonly represented in a simplified two-dimensional phase diagram in which water is omitted and placed at the origin [35].

According to Figure 1a,b, the ability for phase separation of the phosphate salts with the different natural metabolites or NADESs follows the order 3B < 3A~4A < 1B < 1A < 2A. The results suggest that the ATPSs containing NADESs provided a smaller biphasic region than when using their corresponding HBA, independent of the salt employed. This behavior might be due to the difference in hydrophobicity of natural metabolites and NADESs [36,37]. The hydrogen bond acceptors have a similar affinity towards water, which is reduced when they are part of a natural deep eutectic solvent because the interaction with the hydrogen bond donor lessens the number of interactions with water. Then, despite betaine being slightly more hydrophobic than chloride choline, when they are forming DES with urea or levulinic acid, the resulting ATPSs present a very similar binodal curve as the effects compensate [38]. Moreover, as expected, the impact of salt type on the binodal curves follows the salting-out trend predicted by the Hofmeister series [32,33]. Among the systems studied, those containing the salt K_3_PO_4_ showed larger biphasic regions compared to those with K_2_HPO_4_, regardless of the other component used, which agrees with previous studies where K_2_HPO_4_ displayed a strong phase-forming capability [39]. The adjustable parameters calculated using the Merchuk equation (Equation (1)), as well as the coefficient of determination (*R*^2^), were determined to assess the proper fit of the data to the model, and the results are presented in Table 2.

The Merchuk equation adequately fitted the experimental binodal data for the six studied systems. To our knowledge, the literature data are currently available solely for ATPS 1A, 1B, 2A, and 3B, with no reports for 3A and 4A, combining ChCl:2Urea and Bet:2LA used with K_3_PO_4_. Binodal curves obtained in this work exhibit good agreement with the published results for 1A [29], 1B [18], 2A [30], and 3B [31].

#### 2.2.2. Tie-Lines

At equilibrium, the Y and X components partition between the top and bottom phases, and their composition was determined by the tie-lines (TLs), which connect two points on the binodal curve. The behavior of the two-phase region is described by the initial (feed) concentration and the composition of the individual phases at equilibrium, which are fundamental for describing any separation process and understanding how the components of the system behave. Therefore, after determining the binodal curves, several tie-lines were obtained for each ATPS studied based on the experimental compositions of the feeds and the masses of the phases obtained, together with Equations (2)–(5). Two characteristic parameters of the TLs were determined: the tie-line length (TLL) and the tie-line slope (STL), which are commonly used in the study of ATPSs, as they provide relevant information on the amplitude of the two-phase region in the phase diagram [40].

The experimental data of feeds as well as the tie-line compositions determined at 25 °C and atmospheric pressure, along with the calculated length (TLL) and slope (STL) of the tie-lines, are presented in Table 3.

According to previous studies (1A, 1B, 2A, and 3B) [18,29,30,31], and based on experimentally determined binodal curves and tie-lines (2B and 3A), all ATPSs showed a top phase enriched with the component Y, while the bottom phase is enriched with component X of the corresponding ATPS. As can be deduced from Table 3, an increase in TLL leads to an increase in the X concentration in the bottom phase and an increase in the Y concentration in the top phase for all studied systems. The highest values of TLL were obtained for the ATPS 3B {ChCl:2Urea + K_2_HPO_4_ + water}. Regarding the slope of the tie-lines, the differences between the STL values show that the tie-lines are practically parallel, and the steeper slopes were obtained for system 3A. The consistency of the tie-lines was satisfactorily ascertained using the Othmer–Tobias (Equation (8)) and Brancroft (Equation (9)), with coefficients of determination, R^2^, greater than 0.998 (see Supplementary Material).

### 2.3. Protein Extraction with Aqueous Two-Phase Systems

**Figure 2:**
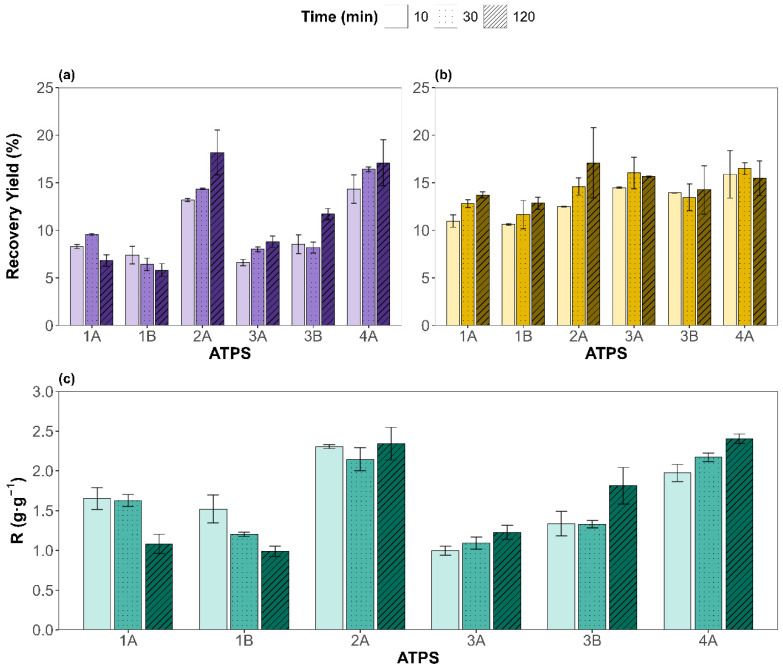
Top-phase results for the recovery yields of (**a**) proteins and (**b**) carbohydrates, in mass percentage; (**c**) depicts the protein-to-carbohydrate mass ratio (R) for each ATPS (x-axis) and different times (different pattern). Experimental data are shown as mean ± SD (n = 2).

The recovery yields for proteins (PRYs) and carbohydrates (CRYs), along with the protein-to-carbohydrate masses ratio (R), were evaluated under different ATPS types and extraction times. The results are shown in Figure 2a, Figure 2b, and Figure 2c, respectively. For the six ATPSs evaluated, protein concentrations extracted from the microalgal biomass to the salt-rich bottom phases were lower than the spectrophotometric detection limit (PRY of 0.9%). For CRYs, the values obtained in the bottom phases were below 5%. Hence, PRYs and CRYs corresponding to the bottom phases have been disregarded and the results discussed hereafter correspond to the protein and carbohydrate recovered in the top phases, rich in component Y (natural metabolite or NADES).

An analysis of variance, ANOVA, of the results (see Supplementary Material) revealed that both factors—type of ATPS and extraction time—were statistically significant (*p* < 0.05) for the PRY and CRY. However, ATPS composition had a dominant effect on the PRY, contributing 85.3% to the total variance versus 2.9% of the extraction time, underscoring the critical role of ATPS components in determining protein partitioning behavior. Interestingly, the interaction between the ATPS and time was also significant (8.5%), suggesting that some systems benefit more from prolonged contact than others, as observed with ATPS 2A, while for 1A, 1B, or 3B it does not have a big impact.

In contrast, for CRYs, the influence of time was more noticeable, with 51.7% of the variance attributed to the ATPS and 12.0% to the extraction time. Still, the pure error contribution accounted for 24.4% of its variance, indicating that the variability of the CRY is less influenced by the factors studied than for the PRY. In addition, the interaction was not significant (*p* = 0.568), supporting the less system-specific behavior in carbohydrate recovery. These results can be attributed to the higher solubility of proteins under alkaline conditions, favoring ATPSs with basic pH (2A and 4A, betaine-based ATPSs), whereas the inherent polarity of carbohydrates renders them less sensitive to variations in ATPS composition. Turning our attention to R, the contribution of the ATPS factor is the largest (82.2%), while the extraction time was non-statistically significant. The remaining variance was mostly due to the ATPS and extraction time interaction (14.7%), supporting the theory that some ATPSs behave differently for distinct extraction times studied. For instance, ATPSs 1A and 1B show higher selectivities for shorter times; 3A, 3B, and 4A display the opposite trend, while 2A remains time independent.

**Figure 3:**
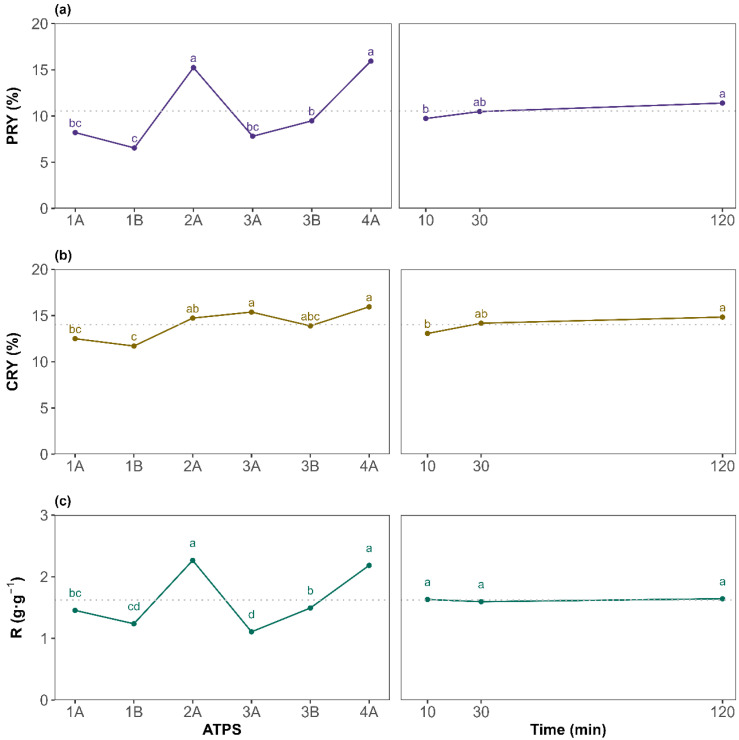
Top-phase mean plots for the different levels of ATPS system (left) and extraction time (right) for (**a**) the protein recovery yield (PRY), (**b**) the carbohydrates recovery yield (CRY), and (**c**) the protein-to-carbohydrate mass ratio (R). The letters on top of each point are displayed according to the compact letter display methodology. Means of the factor levels with a common letter are not significantly different. The horizontal dotted line denotes the great mean of the response.

To assess differences between the tested factor levels, a Tukey HSD *post hoc* test was performed. The results are presented as the mean at each factor level for the PRY, CRY, and R in Figure 3a, Figure 3b, and Figure 3c, respectively. For the PRY, ATPS 2A and 4A (betaine-based ATPSs) yielded, on average, significantly higher values, as indicated by the grouping letters. This could be attributed to betaine, as it acts as a natural protein stabilizer [41]. According to general ATPS behavior, systems with broader biphasic region tend to show a good extraction performance. This agrees with the 2A {Bet + K_3_PO_4_ + water} results as it is the system with the broadest biphasic region, but 4A {Bet:2LA + K_3_PO_4_ + water} showed a narrower biphasic region and gave comparable results to 2A. Hence, the extraction yield is more influenced by the ATPS components than by the binodal curve. For the extraction time, the general upward trend of the PRY with increasing time suggests that sufficient contact between the biomass with the corresponding ATPS is beneficial, although marginal gains beyond 30 min may not justify extended processing times in all cases. Overall, the findings demonstrate that ATPS composition exerts a much greater influence than extraction time, especially for the PRY. Regarding the protein-to-carbohydrate mass ratio (R) (Figure 3c), it presents identical behavior to the PRY when evaluating the effect of each ATPS, with 2A and 4A being the systems providing the highest amount of proteins per carbohydrates co-extracted, although, on average, all systems recovered more proteins than carbohydrates (R greater than 1 g·g^−1^). For the extraction time, as it affected both the PRY and CRY in a similar way, R resulted in a non-significant factor visualized as a horizontal line sharing, for all levels, the same letter a (Figure 3c plot on the right). Therefore, since the main objective of this work is to maximize the PRY while maintaining high selectivity, extending the extraction time to 120 min is not required, on average, for the ATPSs studied. However, as the ATPS and time interaction was significant, attention must be paid to each specific system.

Overall, protein recovery yields ranged from a minimum of 5.82 ± 0.67% (ATPS 1B, 120 min) to a maximum of 18.2 ± 2.3% (ATPS 2A, 120 min). The most promising conditions in terms of selectivity were observed for 4A at 120 min (2.41 ± 0.03 g·g^−1^). Remarkably, ATPS 4A {Bet:2LA + K_3_PO_4_ + water} also delivered a high PRY over shorter extraction times (16.4 ± 0.1% at 30 min), showing no statistical differences with ATPS 2A at 120 min. This suggests potential process time savings with low loss in recovery. Based on these results, ATPS 2A and 4A (betaine-based ATPSs) were identified as the most effective systems. Finally, since extraction time showed no significant effect on selectivity, on average, for all the ATPS studied, 30 min was selected as the optimal duration, offering a satisfactory PRY without substantially increasing the CRY, enabling a faster, more efficient process. The results presented in this study highlight a viable and sustainable alternative to conventional protein recovery methods such as alkaline hydrolysis, or pulsed electric field, which often rely on harsh chemicals and high energy inputs [42]. For instance, Lorenzo-Hernando et al. reported a maximum protein yield of 11.3% from *Scenedesmus almeriensis*, using mild alkaline hydrolysis conditions (2 M NaOH, 40 °C, 30 min) [43]. Rojo et al. also used alkaline and enzymatic hydrolysis for protein extraction from *Scenedesmus almeriensis*; however, their maximum protein-to-carbohydrate ratios ranged between 1 g·g^−1^ (2 M NaOH, 120 °C, 1 h) and 1.7 g·g^−1^ (Alcalase 2.4 AU-A g^−1^, 50 °C, pH 8, 5 h) [44]. These values are lower than those achieved in the present work, despite the use of harsher operating conditions. The higher ratios obtained using the studied NADES-based ATPS indicate enhanced protein selectivity, which can significantly simplify downstream purification and improve the overall industrial viability of the process. Similarly, Safi et al. used a pH of 12, stirring for 2 h at 40 °C, to extract proteins from *Chlorella vulgaris*, getting slightly better results (26%) [45]. In contrast, the studied extraction method achieved a PRY of 16.4% under significantly milder conditions, faster, and using non-toxic, green solvents. Zhang et al. evaluated pulsed electric fields and ultrasonication for extracting both proteins and carbohydrates from different microalgae species (*Nannochloropsis* sp., *P. tricornutum*, and *P. kessleri*), reaching PRY values below 11% and higher carbohydrate recovery yields (CRYs), which resulted in poor selectivity toward protein [46].

Compared to other NADES-based ATPS protein extraction processes, Li et al. showed that a betaine-urea NADES combined with K_2_HPO_4_ salt could extract nearly quantitative amounts of protein (99.82% extraction efficiency) while preserving secondary structure [20]. A similar study using choline chloride together with Na_2_CO_3_ reported high recoveries for both bovine serum albumin (95.16%) and papain (90.95%) under mild conditions, and spectroscopic analyses verified that the protein conformation was unchanged after partitioning [21]. Regarding an application to a complex matrix, Zhu et al. used a betaine-urea/K_2_HPO_4_ NADES-ATPS for walnut protein extraction, delivering a higher overall yield (64.21% vs. 53.32% for the conventional alkali-extraction/isoelectric-precipitation method) [23]. Collectively, these studies illustrate that NADES-based ATPSs constitute a robust, tunable, and environmentally benign platform for the extraction and purification of proteins across a wide spectrum of biotechnological applications, but their application to real complex matrices like microalgal biomass is still scarce. The most notable application to microalgae is the work by Menegotto et al. [47], who designed an ATPS process for protein recovery from *Arthrospira platensis*, achieving 68.7–80.3% yields. However, their protocol involved alkaline pretreatment (2 M NaOH), ultrasonication, mechanical stirring (50 min), and operation at 30 °C.

Thus, the method presented in this study offers some advantages in terms of process simplicity, safety, and environmental compatibility. In addition, it is also worth highlighting that the most effective systems, 2A and 4A (ATPSs involving betaine), contain 50% water. This high aqueous content reinforces the green and sustainable character of the proposed process, minimizing the use of organic solvents while maintaining the extraction performance. Nevertheless, some limitations must be acknowledged. Protein recovery yields obtained in a single extraction step, although achieved under significantly milder conditions and in shorter times, remain moderate compared to multi-step or strongly alkaline processes. Despite these limitations, the results demonstrate the potential of green ATPS systems as a promising platform for selective protein recovery from complex microalgal matrices within a sustainable biorefinery framework.

## 3. Materials and Methods

### 3.1. Biomass Cultivation and Characterization

The biomass used in this work was provided by the Cajamar Experimental Station, University of Almería (Spain), and consisted of a consortium of microalgae and bacteria grown in a 1200 L thin-layer photobioreactor with a dilution rate of 0.33 d^−1^ and fed with 10% diluted swine wastewater [48]. A single batch of this biomass was centrifuged, freeze-dried, and well mixed to ensure a homogeneous composition. According to previous research, *Scenedesmus almeriensis* was the most abundant microalgae species in the consortium (96%) [49]. The lyophilized biomass was stored at 4 °C until further use to prevent degradation. Standard methods were used to quantify the main components of the biomass, including water and ash (AOAC 942.05 method) [50], proteins (Kjeldahl method) [51], carbohydrates (based on phenol-sulfuric acid method) [52], and lipids (Folch method) [53]. The chemical composition of the lyophilized biomass was 44.5% protein, 20.4% carbohydrates, 11.9% lipids, 15.1% ash, and 5.6% water.

### 3.2. Aqueous Two-Phase Systems

#### 3.2.1. ATPS Screening

As a preliminary study, the formation of several ATPSs was experimentally tested at 25 °C and atmospheric pressure through the combination of three components: a natural metabolite or a NADES (1), salting-out agent (2), and water (3). As natural metabolites were used: choline chloride (ChCl), betaine (Bet), urea, and levulinic acid (LA), and two NADESs combining ChCl with urea (ChCl:2Urea) and betaine with levulinic acid (Bet:2LA) in a 1:2 molar ratio. The NADESs were prepared by mixing the corresponding HBA and HBD and heating the mixture to 60 ± 0.1 °C under continuous stirring using an orbital incubator (Optic Ivymen System, Logroño, Spain). Upon formation of a homogeneous, transparent liquid, the mixture was allowed to cool to room temperature and stored for further use. To prepare the ATPSs, aqueous solutions of natural metabolites (ChCl, Bet, urea, or LA), or NADESs (ChCl:2Urea or Bet:2LA), and salts were used. Aqueous solutions of ChCl, Bet, and urea were necessary since both compounds are solid at room temperature and tend to dissolve slowly when added directly to a mixture. Aqueous solutions of NADESs were employed to facilitate handling, given their high viscosity, while keeping a high concentration. The salt solutions were prepared at compositions close to their maximum solubility in water at 25 °C to ensure a homogeneous mixture. The tests were performed by gradually adding an aqueous solution of the natural metabolite or of the NADES (1) to the concentrated aqueous solution of the selected salt (2) until turbidity was observed. The composition of each mixture was determined by weighing the individual components. The concentration of the aqueous solutions (in mass percentage) for each compound was ChCl (80.0%), Bet (56.5%), Urea (49.0%), LA (98.0%), ChCl:2Urea (85.6%), Bet:2LA (80.0%), K_3_PO_4_ (49.0%), K_2_HPO_4_ (60.5%), Na_3_C_6_H_5_O_7_ (30.6%), Na_2_C_4_H_4_O_6_ (26.9%), and KNaC_4_H_4_O_6_ (30.0%). These aqueous solutions were used throughout the experimental determinations carried out in this work. The phosphate salts were acquired from Merck (Darmstadt, Germany), sodium citrate tribasic dihydrate, disodium L-(+)-tartrate dihydrate, and potassium sodium tartrate tetrahydrate were acquired from Sigma-Aldrich (Darmstadt, Germany). Choline chloride, betaine, urea, and levulinic acid, were purchased from Fisher Scientific (Waltham, MA, USA). All chemicals were of analytical grade and used without further purification.

#### 3.2.2. Phase Diagrams of ATPS

Based on the screening results and on the literature research, six ATPSs were selected to carry out the experiments reported in this work. Table 4 summarizes the ATPSs assayed {Component Y (1) + Component X (2) + water (3)}, and the code assigned to each solvent to simplify the discussion of the results, where the number refers to component Y: 1 for ChCl, 2 for Bet, 3 for NADES ChCl:2Urea, and 4 for NADES Bet:2LA, and the letter refers to the component X: A for K_3_PO_4_ and B for K_2_HPO_4_.

The fundamental concept of ATPSs is that they are ternary systems in which water is the main component. By combining the aqueous solution of natural metabolite or NADES with salt in adequate proportions, two immiscible liquid phases are formed [35]. One of the phases (top phase) is rich in one component, while the bottom phase is enriched in the other.

##### Binodal Curves Determination

Binodal curves were built to define the boundary between the monophasic and biphasic regions of the six ternary mixtures assayed. Binodal curves were experimentally obtained at 25 °C and atmospheric pressure using the cloud point titration method [54]. A known amount of the salt aqueous solution (K_3_PO_4_ or K_2_HPO_4_) was introduced into glass tubes of 15 mL placed in a thermostated bath. The aqueous solution of the natural metabolite (ChCl or Bet) or NADES (ChCl:2Urea or Bet:2LA) was added dropwise and stirred until permanent slight turbidity in the mixtures was observed. At this point, the composition of the mixtures was determined by weighing. Afterwards, drops of deionized water were added until a homogeneous mixture was achieved again, and the above procedure was repeated in the same glass tube until enough data were collected. To cover the entire composition range of the binodal curve, the process was repeated in reverse by adding amounts of the salt solution to a known amount of the natural metabolite or NADES solution, following the aforementioned procedure. These experimental data were merged to build the complete binodal curve. Binodal curves were fitted using the equation proposed by Merchuck et al. (Equation (1)) [55], which is widely employed for ATPSs [35]:

(1) $$w_{Y}=A\cdot \exp\left[B\cdot w_{X}^{0.5}-C\cdot w_{X}^{3}\right]$$

where $w_{Y}$ and $w_{X}$ are the compositions of the ATPS components X and Y in the binodal curve in mass fraction, respectively, and A, B, and C are the adjustable parameters obtained by nonlinear least squares regression.

##### Tie-Lines Determination

The experimental determination of tie-lines for the six studied ATPSs was carried out at 25 °C and atmospheric pressure. Immiscible mixtures of known compositions of the corresponding components were prepared by weighing in glass tubes and sealed with silicone caps to prevent losses due to evaporation or moisture absorption. These mixtures were placed in a thermostatic bath at 25 °C, where they were vigorously stirred to ensure thorough mixing. After agitation, the samples were maintained at the same temperature until complete phase separation was achieved. Subsequently, both phases were carefully separated using syringes and individually weighed.

TL compositions were obtained using the gravimetric method originally proposed by Merchuk et al. [56], which has also been used for ATPSs based on DES [31]. To determine the compositions of both equilibrium phases, a system of four equations with four unknowns needs to be solved. The unknowns are the concentrations of Y and X in the top, $w_{Y(Top)}$ and $w_{X(Top)}$, and bottom phases, $w_{Y(Bot)}$ and $w_{X(Bot)}$. The known quantities are the overall composition of the mixture, $w_{Y(Mix)}$ and $w_{X(Mix)}$, and the phases’ mass ratio, $\alpha =\frac{m_{\left(Top\right)}}{m_{\left(Bot\right)}}$, where $m_{\left(Top\right)}$ is the mass of the top phase, and $m_{\left(Bot\right)}$ is the mass of the bottom phase, and the parameters of the Merchuk equation (A, B, and C) are previously determined by nonlinear regression fit from binodal data (Equation (1)). Thus, for each phase, the same composition variable is described by two independent relations: one is derived from the Merchuk binodal equation and another from the overall mass balance [55]. Therefore, Equations (2)–(5) constitute a coupled system that must be solved simultaneously to obtain the equilibrium phase compositions:

(2) wYTop=A· expB·wX Top0.5−C·wX Top3

(3) wYBot=A·expB·wX Bot0.5−C·wX Bot3

(4) $$w_{Y(Top)}=\frac{1}{\alpha }\cdot w_{Y(\mathrm{Mix})}-\frac{1-\alpha }{\alpha }\cdot w_{Y(\mathrm{Bot})}$$

(5) $$w_{X(Top)}=\frac{1}{\alpha }\cdot w_{X(\mathrm{Mix})}-\frac{1-\alpha }{\alpha }\cdot w_{X(\mathrm{Bot})}$$

This nonlinear system was solved using numerical methods, and the results give the mass fraction composition of Y and X in both phases.

The characteristic parameters of the TL, the tie-lines length (TLL), and the tie-line slopes (STL), were calculated using Equations (6) and (7):

(6) $$TLL=\sqrt{{\left[w_{Y\left(Top\right)}-w_{Y\left(Bot\right)}\right]}^{2}+{\left[w_{X\left(Top\right)}-w_{X\left(Bot\right)}\right]}^{2}}$$

(7) $$STL=\frac{w_{Y\left(Top\right)}-w_{Y\left(Bot\right)}}{w_{X\left(Top\right)}-w_{X\left(Bot\right)}}$$

In addition, the degree of consistency of the tie-lines data was tested using the equations proposed by Othmer–Tobias (Equation (8)) and Brancroft (Equation (9)), which are the most widely applied for ATPSs [56,57].

(8) $$\log\left[\frac{1-w_{Y\left(Top\right)}}{w_{Y\left(Top\right)}}\right]=K_{1}+n\cdot \log\left[\frac{1-w_{X\left(Bot\right)}}{w_{X\left(Bot\right)}}\right]$$

(9) $$\log\left[\frac{1-w_{water\left(Bot\right)}}{w_{X\left(Bot\right)}}\right]=K_{2}+r\cdot \log\left[\frac{1-w_{water\left(Top\right)}}{w_{Y\left(Top\right)}}\right]$$

where $w_{water\left(Top\right)}$ and $w_{water\left(Bot\right)}$ account for the mass fraction of water in the top and bottom phase, respectively, and $K_{1}$ and n are the characteristic adjustable parameters from Othmer–Tobias model, while $K_{2}$ and r, are the adjustable parameters for the Brancroft equation.

### 3.3. Extraction Experiments

Extraction experiments were performed to evaluate the influence of the type of ATPS used and the extraction time to maximize protein recovery while minimizing carbohydrate co-extraction as carbohydrates are the second major fraction in the employed microalgal biomass. For this purpose, a two-factor full factorial design was used to assess the significant effects of the factors (type of ATPS and extraction time). All experiments were carried out in duplicate to assess the interaction between the factors and to achieve a good estimation of the residual error. The type of ATPS is also a critical factor in the extraction process as it may condition its performance and sustainability; therefore, six different ATPSs were used (1A, 1B, 2A, 3A, 3B, and 4A, see Table 4). To our knowledge, none of the studied ATPSs have been applied for protein extraction in microalgae matrices. Extraction time was studied as an important operational parameter of any separation procedure; thus, three different values were chosen (10, 30, and 120 min) to assess its influence on recovery efficiency. Although longer extraction times may enhance partitioning due to improved phase equilibration or increased molecular diffusion, shorter extraction times are generally preferable from a process efficiency standpoint if extraction yield is not significantly compromised.

The extraction experiments were performed on the selected tie-line for each of the systems studied at 25 °C. For this, 4.5 g of feed compositions within immiscible regions of each ATPS were gravimetrically prepared in glass tubes by mixing the appropriate amounts of each component. Then, approximately 30 mg of freeze-dried microalgae, manually disaggregated using a manual mortar, was added to each ATPS. The ratio biomass: ATPS was set according to similar values found in the literature [58,59]. Next, mixtures were stirred and placed in a thermostatic bath at 25 °C, a temperature selected both for thermodynamic consistency and for its relevance to energy-efficient industrial operation. Then, they were left to settle down for a given time (10, 30, or 120 min) to ensure an adequate phase splitting. After the corresponding time, the two phases were separated using syringes, filtered through 0.45 μm Nylon filters into 25 mL volumetric flasks, and diluted to volume with deionized water for subsequent quantification of protein and carbohydrates in each extract.

### 3.4. Quantification of Proteins and Carbohydrates

Spectrophotometric methods were used to quantify the analytes of interest. Protein concentration was determined using the bicinchoninic acid (BCA) assay kit supplied by Fisher Scientific (Hampton, NH, USA), following the manufacturer’s instructions. A calibration curve was prepared using bovine serum albumin (BSA) as standard protein [60]. For carbohydrate analysis, the phenol-sulfuric acid method was applied [61], with D-(+)-glucose used as standard to construct the calibration curve, allowing total carbohydrate content to be expressed as glucose equivalents [62]. D-glucose was obtained from Sigma Aldrich (Burlington, VT, USA) and phenol from Merck (Darmstadt, Germany), while sulfuric acid was provided by PanReac (Barcelona, Spain). All reagents used were of analytical grade and applied without further purification. Absorbance measurements were carried out in duplicate using a Spectronic Genesys 5 spectrophotometer (Waltham, MA, USA). All determinations were carried out in duplicate. Extraction performance was evaluated through the protein recovery yield (*PRY*) and carbohydrate recovery yield (*CRY*), both expressed as a percentage with respect to the content of each analyte studied in the initial biomass. These were calculated according to Equations (10) and (11):

(10) $$PRY=\frac{{V\cdot c}_{P}}{m_{0}\cdot \;P}\cdot 100$$

(11) $$CRY=\frac{{V\cdot c}_{C}}{m_{0}\cdot C}\cdot 100$$

where V (L) is the final volume to which the separated phase of each ATPS was diluted, $c_{P}$ and $c_{C}$ (mg·L^−1^) are the protein and carbohydrate concentrations in the diluted extract, $m_{0}$ is the amount of biomass (mg), and P and C are the mass fractions of protein and carbohydrate in the biomass, respectively.

Since the protein content in the biomass is double that of the carbohydrates, defining a ratio between the extracted protein and carbohydrate masses provides a clearer understanding of the process selectivity. For this purpose, a protein-to-carbohydrate mass ratio variable (*R*) was defined:

(12) $$R=\frac{m_{p}}{m_{c}}=\frac{{V\cdot c}_{P}}{V\cdot c_{c}}=\frac{PRY\cdot P}{CRY\cdot C}$$

where $m_{p}$ is the mass of proteins extracted and $m_{c}$ the mass of carbohydrates recovered, in mg.

### 3.5. Statistical Analysis

Data are presented as mean ± standard deviation (SD). Analysis of variance (ANOVA) was carried out to test the significance of the two factors for each response variable of the extraction experiments. Tukey’s HSD *post hoc* test was used to assess statistically significant differences between the levels of each factor. The compact letter display (cld) method was employed to succinctly present the results of multiple comparisons [63,64]. All statistical analyses were performed at the 5% significance level (α = 0.05). Statistical analysis and graph generation were conducted using R software (version 4.5.1).

## 4. Conclusions

In this work, six aqueous two-phase systems (ATPSs) were prepared using different combinations of natural deep eutectic solvents (NADESs) or natural metabolites with phosphate salts. Binodal curves and tie-lines were obtained to characterize each system. The ability of phase separation varied across systems, and the ones based on NADESs exhibited narrower biphasic regions, regardless of the salt used. The six ATPSs were subsequently applied to the extraction of protein from microalgae *Scenedesmus almeriensis* grown in piggery wastewater, testing three different times. The results showed that the ATPS components had a more significant impact on protein recovery yield (PRY) than biphasic region size and extraction time, for which 30 min was sufficient to reach a good compromise between protein recovery and selectivity towards carbohydrates. Systems 2A and 4A (based on betaine) achieved the best results (18.2% at 120 min and 16.4% at 30 min, respectively), which are comparable to values reported for conventional extraction methods such as mild alkaline hydrolysis, and the highest selectivity relative to carbohydrate extraction (2.17 g·g^−1^). This system contains up to 50% water, highlighting the environmentally friendly character of the process and its reduced dependence on harmful or volatile solvents. These findings demonstrate that the selection of phase-forming components is key for optimizing extraction efficiency and highlights the potential of natural-based ATPSs as a tunable and sustainable separation alternative for biomolecule separation.

## Figures and Tables

**Table 1:** Study of the ATPS formation with aqueous solutions of selected natural metabolites or NADES and salts at 25 °C and atmospheric pressure.

Natural Compound or DES	Salt				
	K_3_PO_4_	K_2_HPO_4_	Na_3_C_6_H_5_O_7_	KNaC_4_H_4_O_6_	KNaC_4_H_4_O_6_
**ChCl**	✓ ^a,^* [29]	✓ ^a,^* [18]	Precipitation	Precipitation	✗
**Bet**	✓ ^a,^* [30]	✓ * [30]	✗	✗	✗
**ChCl:2Urea**	✓ ^a^	✓ ^a^ [31]	Precipitation	Precipitation	✗
**Bet:2LA**	✓ ^a^	✗	✗	✗	✗
**Urea**	✗	✗	✗	✗	✗
**LA**	Precipitation	Precipitation	Precipitation	Precipitation	Precipitation

✓ Means two immiscible liquid phases can be formed. ✗ Means two immiscible liquid phases did not form. Precipitation indicates homogeneous liquid phase with a precipitated component. ^a^ Denotes ATPS experimental data presented in this work. * Indicates ATPS experimental data previously reported and its reference.

**Table 2:** Adjustable parameters (A, B, and C) obtained by Merchuk equation (Equation (1)) and the coefficient of determination (*R*^2^) for the experimental data of the binodal curves.

Merchuk’s Adjustable Parameters				
ATPS	A	B	C	*R* ^2^
1A	0.901	−2.534	23.49	0.9995
1B	0.815	−2.168	12.52	0.9998
2A	0.678	−2.371	31.45	0.9998
3A	1.163	−2.224	19.10	0.9997
3B	1.268	−2.007	10.95	0.9997
4A	1.059	−1.883	26.37	0.9997

**Table 3:** Tie-line compositions (in mass fraction) for the feed, top, and bottom phases of the top phase component ($w_{Y}$) and bottom phase component ($w_{X}$), together with their corresponding tie-line slope (STL) and length (TLL), for each ATPS at 25 °C and atmospheric pressure.

Tie-Lines	Feed		Top Phase		Bottom Phase		STL	TLL
	$w_{Y}$	$w_{X}$	$w_{Y}$	$w_{X}$	$w_{Y}$	$w_{X}$		
**1A**								
**TL 1**	0.225	0.226	0.347	0.128	0.073	0.348	−1.25	0.35
**TL 2**	0.104	0.350	0.434	0.081	0.036	0.405	−1.23	0.51
TL 3 *	0.200	0.349	0.595	0.026	0.006	0.508	−1.22	0.76
**1B**								
**TL 1**	0.272	0.245	0.465	0.066	0.072	0.431	−1.08	0.54
**TL 2**	0.296	0.301	0.635	0.013	0.026	0.530	−1.18	0.80
TL 3 *	0.199	0.397	0.656	0.010	0.021	0.548	−1.18	0.83
**2A**								
**TL 1**	0.151	0.251	0.261	0.136	0.029	0.378	−0.96	0.34
**TL 2**	0.300	0.198	0.513	0.014	0.008	0.450	−1.16	0.67
**TL 3**	0.196	0.300	0.542	0.009	0.006	0.459	−1.19	0.70
TL 4 *	0.154	0.345	0.565	0.006	0.005	0.468	−1.21	0.73
**3A**								
**TL 1**	0.377	0.221	0.615	0.080	0.097	0.386	−1.69	0.60
**TL 2**	0.199	0.350	0.659	0.064	0.051	0.442	−1.61	0.72
TL 3 *	0.250	0.349	0.702	0.051	0.023	0.498	−1.52	0.81
**3B**								
**TL 1**	0.303	0.349	0.658	0.103	0.071	0.510	−1.44	0.72
TL 2 *	0.393	0.307	0.738	0.072	0.053	0.538	−1.47	0.83
**TL 3**	0.355	0.378	0.870	0.035	0.026	0.597	−1.50	1.01
**4A**								
**TL 1**	0.196	0.332	0.472	0.148	0.031	0.442	−1.50	0.53
TL 2 *	0.301	0.298	0.664	0.060	0.014	0.487	−1.52	0.78
**TL 3**	0.348	0.294	0.752	0.033	0.008	0.513	−1.55	0.89

* An asterisk indicates the TL where the extraction experiment was carried out.

**Table 4:** ATPSs tested and their coding according to their components involved in each system {Component Y (1) + Component X (2) + water (3)}.

ATPS Code	ATPS Components
1A	ChCl + K_3_PO_4_ + water
1B	ChCl + K_2_HPO_4_ + water
2A	Bet + K_3_PO_4_ + water
3A	ChCl:2Urea + K_3_PO_4_ + water
3B	ChCl:2Urea + K_2_HPO_4_ + water
4A	Bet:2LA + K_3_PO_4_ + water

## Data Availability

The raw data supporting the conclusions of this article will be made available by the authors on request.

## Supplementary Materials

The following supporting information can be downloaded at: https://www.mdpi.com/article/10.3390/molecules31030483/s1, Table S1: Experimental binodal data for the ATPS studied. $w_{Y}$ indicates the mass fraction of the top phase constituent and $w_{X}$ the mass fraction of the bottom phase constituent used; Table S2: Othmer–Tobias and Brancroft parameters and their coefficients of determination. Determination coefficients were higher than 0.998 for all ATPS; Table S3: Full factorial design experiments for the extraction of *Scenedesmus almeriensis* proteins. The data for each treatment is expressed as mean ± standard deviation of two replicates for the three response variables (PRY, CRY, and R); and Table S4: Contribution to the total variance and *p*-values of the factors and factor interaction from the full factorial design for the response variables protein recovery yield (PRY), carbohydrates recovery yield (CRY), and the extracted protein-to-carbohydrate ratio (R). Significant factors highlighted in red.

## Abbreviations

The following abbreviations are used in this manuscript:

ATPS	Aqueous Two-Phase Systems
DES	Deep Eutectic Solvents
HBA	Hydrogen Bond Acceptors
HBD	Hydrogen Bond Donors
NADES	Natural Deep Eutectic Solvents
ChCl	Choline Chloride
Bet	Betaine
LA	Levulinic Acid
PRY	Protein Recovery Yield
CRY	Carbohydrate Recovery Yield
R	Protein-To-Carbohydrate Mass Ratio
SD	Standard Deviation
ANOVA	Analysis of Variance
