# Supplementary material for: Mild One-Step Protein Recovery from Microalgae Cultivated in Swine Wastewater Using Natural Deep Eutectic Solvent-Based Aqueous Biphasic Systems
Source: Molecules. 2026 Jan 30;31(3):483. doi: 10.3390/molecules31030483 (PMC12899697; doi:10.3390/molecules31030483)
Supplement: Supplementary file 1 [file molecules-31-00483-s001.zip › molecules-4094575-supplementary.pdf]

SUPPLEMENTARY DATA -

Table S1: Experimental binodal data for the ATPS studied. w_Y indicates de mass fraction of the top phase constituent, and w_X the mass fraction of the bottom phase constituent used.

ATPS											
**1A**		**1B**		**2A**		**3A**		**3B**		**4A**	
w_Y	w_X	w_Y	w_X	w_Y	w_X	w_Y	w_X	w_Y	w_X	w_Y	w_X
0.019	0.479	0.007	0.600	0.004	0.487	0.053	0.448	0.034	0.575	0.020	0.461
0.024	0.452	0.014	0.563	0.011	0.450	0.078	0.408	0.035	0.584	0.071	0.381
0.033	0.412	0.019	0.543	0.062	0.320	0.099	0.379	0.042	0.564	0.090	0.37
0.037	0.395	0.024	0.520	0.089	0.288	0.149	0.337	0.048	0.560	0.111	0.352
0.054	0.367	0.037	0.502	0.118	0.259	0.218	0.292	0.051	0.538	0.120	0.346
0.074	0.348	0.045	0.479	0.147	0.231	0.249	0.273	0.059	0.516	0.129	0.339
0.097	0.318	0.057	0.458	0.172	0.210	0.288	0.249	0.077	0.503	0.170	0.309
0.110	0.309	0.074	0.431	0.194	0.190	0.311	0.235	0.112	0.458	0.208	0.281
0.120	0.298	0.091	0.405	0.226	0.165	0.335	0.220	0.122	0.443	0.148	0.326
0.126	0.291	0.096	0.396	0.235	0.156	0.357	0.207	0.128	0.438	0.170	0.308
0.130	0.288	0.106	0.383	0.245	0.148	0.380	0.194	0.152	0.419	0.386	0.194
0.158	0.266	0.110	0.377	0.256	0.140	0.408	0.176	0.156	0.422	0.441	0.161
0.175	0.256	0.115	0.371	0.269	0.130	0.433	0.165	0.171	0.403	0.495	0.134
0.183	0.246	0.128	0.351			0.446	0.158	0.184	0.396	0.549	0.108
0.213	0.223	0.139	0.343			0.467	0.145	0.205	0.382	0.595	0.089
0.227	0.212	0.144	0.338			0.534	0.113	0.220	0.367	0.645	0.070
0.237	0.204	0.162	0.316			0.571	0.098	0.266	0.336	0.702	0.046
0.250	0.194	0.172	0.306					0.312	0.303		
0.259	0.187	0.180	0.298					0.348	0.280		
0.273	0.177	0.186	0.292					0.380	0.259		
0.291	0.161	0.194	0.284					0.405	0.243		
0.304	0.153	0.245	0.234					0.431	0.226		
0.327	0.138	0.265	0.215					0.460	0.208		
0.335	0.132	0.271	0.207					0.471	0.200		
0.356	0.120	0.283	0.197					0.478	0.195		
0.381	0.110	0.290	0.190					0.484	0.192		
0.424	0.087	0.313	0.168					0.492	0.188		
0.436	0.079	0.337	0.150					0.556	0.154		
0.455	0.071	0.368	0.124					0.573	0.147		
0.468	0.064	0.424	0.087					0.590	0.137		
0.485	0.059	0.436	0.079					0.657	0.106		
0.525	0.046	0.455	0.071					0.691	0.090		
		0.463	0.066					0.721	0.077		

<u>Table S2</u>: Othmer-Tobias and Brancroft parameters and their coefficients of determination. Determination coefficients were higher than 0.998 for all ATPS.

ATPS	Othmer-Tobias		Bancroft	
	n	***K_1***	***r***	***K_2***
1A	1.538	-0.144	0.644	0.107
1B	1.694	-0.144	0.550	0.060
2A	3.561	-0.323	0.089	0.296
3A	0.854	-0.374	0.956	0.418
3B	3.534	-0.220	0.220	0.011
4A	4.310	-0.387	0.234	0.098

Table S3: Full factorial design experiments for the extraction of *Scenedesmus almeriensis* proteins. The data for each treatment is expressed as mean ± standard deviation of two replicates for the three response variables (PRY, CRY, and R).

**Trial**	**ATPS**	**Extraction time (min)**	**PRY (%)**	**CRY (%)**	**R ($g \cdot g^{-1}$)**
1	1A {ChCl + K_3PO_4 + water}	10	8.29 ± 0.21	11.0 ± 0.6	1.65 ± 0.06
2	1A {ChCl + K_3PO_4 + water}	30	9.55 ± 0.12	12.81 ± 0.42	1.63 ± 0.03
3	1A {ChCl + K_3PO_4 + water}	120	6.8 ± 0.6	13.71 ± 0.31	1.08 ± 0.06
4	1B {ChCl + K_2HPO_4 + water}	10	7.4 ± 0.9	10.6 ± 0.1	1.52 ± 0.08
5	1B {ChCl + K_2HPO_4 + water}	30	6.4 ± 0.7	11.6 ± 1.5	1.20 ± 0.01
6	1B {ChCl + K_2HPO_4 + water}	120	5.8 ± 0.7	12.8 ± 0.6	0.99 ± 0.03
7	2A {Bet + K_3PO_4 + water}	10	13.20 ± 0.16	12.5 ± 0.01	2.31 ± 0.01
8	2A {Bet + K_3PO_4 + water}	30	14.3 ± 0.1	14.6 ± 0.9	2.15 ± 0.07
9	2A {Bet + K_3PO_4 + water}	120	18.2 ± 2.3	17.1 ± 3.7	2.34 ± 0.09
10	3A {ChCl:2Urea + K_3PO_4 + water}	10	6.62 ± 0.33	14.5 ± 0.1	1.00 ± 0.03
11	3A {ChCl:2Urea + K_3PO_4 + water}	30	8.00 ± 0.26	16.0 ± 1.6	1.09 ± 0.04
12	3A {ChCl:2Urea + K_3PO_4 + water}	120	8.8 ± 0.6	15.6 ± 0.1	1.23 ± 0.04
13	3B {ChCl:2Urea + K_2HPO_4 + water}	10	8.5 ± 0.9	13.9 ± 0.1	1.34 ± 0.07
14	3B {ChCl:2Urea + K_2HPO_4 + water}	30	8.2 ± 0.6	13.5 ± 1.4	1.33 ± 0.02
15	3B {ChCl:2Urea + K_2HPO_4 + water}	120	11.7 ± 0.6	14.2 ± 2.5	1.81 ± 0.11
16	4A {Bet:2LA + K_3PO_4 + water}	10	14.3 ± 1.5	15.9 ± 2.5	1.97 ± 0.05
17	4A {Bet:2LA + K_3PO_4 + water}	30	16.4 ± 0.24	16.5 ± 0.6	2.17 ± 0.02
18	4A {Bet:2LA + K_3PO_4 + water}	120	17.1 ± 2.4	15.5 ± 1.8	2.41 ± 0.03

Table S4: Contribution to the total variance and *p*-values of the factors and factor interaction from the full factorial design for the response variables protein recovery yield (PRY), carbohydrates recovery yield (CRY), and the extracted protein-to-carbohydrate ratio (R). Significant factors highlighted in red.

	PRY		CRY		R	
**Factor**	***Contribution / %***	***p-value***	***Contribution / %***	***p-value***	***Contribution / %***	***p-value***
ATPS	85.3	0.0000	51.7	0.0000	82.2	0.0000
Time	2.9	0.0027	12.0	0.0271	0.2	0.5960
ATPS – Time interaction	8.5	0.0018	11.9	0.5681	14.7	0.0000
